# Accuracy of Artificial Intelligence–Based Automated Quantitative Coronary Angiography Compared to Intravascular Ultrasound: Retrospective Cohort Study

**DOI:** 10.2196/45299

**Published:** 2023-04-26

**Authors:** In Tae Moon, Sun-Hwa Kim, Jung Yeon Chin, Sung Hun Park, Chang-Hwan Yoon, Tae-Jin Youn, In-Ho Chae, Si-Hyuck Kang

**Affiliations:** 1 Uijeongbu Eulji University Hospital Uijeongbu Republic of Korea; 2 Seoul National University Bundang Hospital Seongnam Republic of Korea

**Keywords:** artificial intelligence, AI, coronary angiography, coronary stenosis, interventional ultrasonography, coronary, machine learning, angiography, stenosis, automated analysis, computer vision

## Abstract

**Background:**

An accurate quantitative analysis of coronary artery stenotic lesions is essential to make optimal clinical decisions. Recent advances in computer vision and machine learning technology have enabled the automated analysis of coronary angiography.

**Objective:**

The aim of this paper is to validate the performance of artificial intelligence–based quantitative coronary angiography (AI-QCA) in comparison with that of intravascular ultrasound (IVUS).

**Methods:**

This retrospective study included patients who underwent IVUS-guided coronary intervention at a single tertiary center in Korea. Proximal and distal reference areas, minimal luminal area, percent plaque burden, and lesion length were measured by AI-QCA and human experts using IVUS. First, fully automated QCA analysis was compared with IVUS analysis. Next, we adjusted the proximal and distal margins of AI-QCA to avoid geographic mismatch. Scatter plots, Pearson correlation coefficients, and Bland-Altman were used to analyze the data.

**Results:**

A total of 54 significant lesions were analyzed in 47 patients. The proximal and distal reference areas, as well as the minimal luminal area, showed moderate to strong correlation between the 2 modalities (correlation coefficients of 0.57, 0.80, and 0.52, respectively; *P*<.001). The correlation was weaker for percent area stenosis and lesion length, although statistically significant (correlation coefficients of 0.29 and 0.33, respectively). AI-QCA tended to measure reference vessel areas smaller and lesion lengths shorter than IVUS did. Systemic proportional bias was not observed in Bland-Altman plots. The biggest cause of bias originated from the geographic mismatch of AI-QCA with IVUS. Discrepancies in the proximal or distal lesion margins were observed between the 2 modalities, which were more frequent at the distal margins. After the adjustment of proximal or distal margins, there was a stronger correlation of proximal and distal reference areas between AI-QCA and IVUS (correlation coefficients of 0.70 and 0.83, respectively).

**Conclusions:**

AI-QCA showed a moderate to strong correlation compared with IVUS in analyzing coronary lesions with significant stenosis. The main discrepancy was in the perception of the distal margins by AI-QCA, and the correction of margins improved the correlation coefficients. We believe that this novel tool could provide confidence to treating physicians and help in making optimal clinical decisions.

## Introduction

Coronary angiography is a key step in defining the coronary anatomy and severity of coronary arterial stenosis [1]. Percent diameter stenosis (%DS) based on a 2D image is usually used as evidence of ischemia or guidance for further physiology study [2]. Despite advances in intravascular imaging and physiology, coronary intervention is mostly performed based on coronary angiography alone [3].

Efforts have been made to analyze coronary angiography images quantitatively and objectively [4]. Human eyeball assessments are known to have a high interobserver variability [5]. Quantitative coronary angiography (QCA) has proven reproducibility and accuracy and is thus considered the standard [6]. Moreover, 3D QCA has been developed, which showed a better correlation with coronary hemodynamics and intravascular anatomy than the 2D QCA [7,8]. However, its clinical adoption is low because it is time consuming and labor intensive.

Intravascular ultrasound (IVUS) offers detailed 3D tomographic views of coronary plaques and reference vessels. Anatomical information obtained by IVUS can help identify the clinical relevance of the lesion and enable optimal stent implantation [9]. Studies have suggested that the use of IVUS can reduce adverse cardiovascular events such as mortality, myocardial infarction, target lesion revascularization, and stent thrombosis, especially in complex coronary interventions, including left main intervention and long coronary stenting [10-12]. Limitations of intravascular imaging still exist, such as the additional time and cost as well as the invasiveness of the additional procedure.

Artificial intelligence (AI) has been shown to automatically analyze medical images with accuracy and consistency as high as human experts [13]. A novel software (MPXA-2000, Medipixel) has been developed that uses a deep learning algorithm to segment and analyze coronary angiography images. An AI-assisted real-time QCA that automatically provides quantitative information has the potential to support clinical decisions and improve patient outcomes. In this study, we validated the performance of AI-based QCA (AI-QCA) compared with IVUS in patients with coronary artery disease.

## Methods

### Study Design and Patient Selection

This was a retrospective analysis of patients with coronary artery disease who underwent coronary intervention at a single tertiary center. Fifty patients who underwent IVUS-guided percutaneous coronary intervention (PCI) in Uijeongbu Eulji University Hospital between October 2021 and July 2022 were included. Patients with total or subtotal occlusion and ST-segment elevation myocardial infarction were excluded from the study. Baseline characteristics, clinical diagnosis, and laboratory data were collected via medical record review.

### Ethical Considerations

The study protocol was approved by the Eulji University Hospital Institutional Review Board (no. 2022-07-009). Written informed consent was waived because of the retrospective study design and minimal risk to the patients. Personal information and study data were anonymous and deidentified. The data will not be used for any purpose other than this research, and compensation for participants is not applicable. The study complied with the principles of the Declaration of Helsinki, revised in 2013.

### AI-QCA Analysis

**Figure 1:**
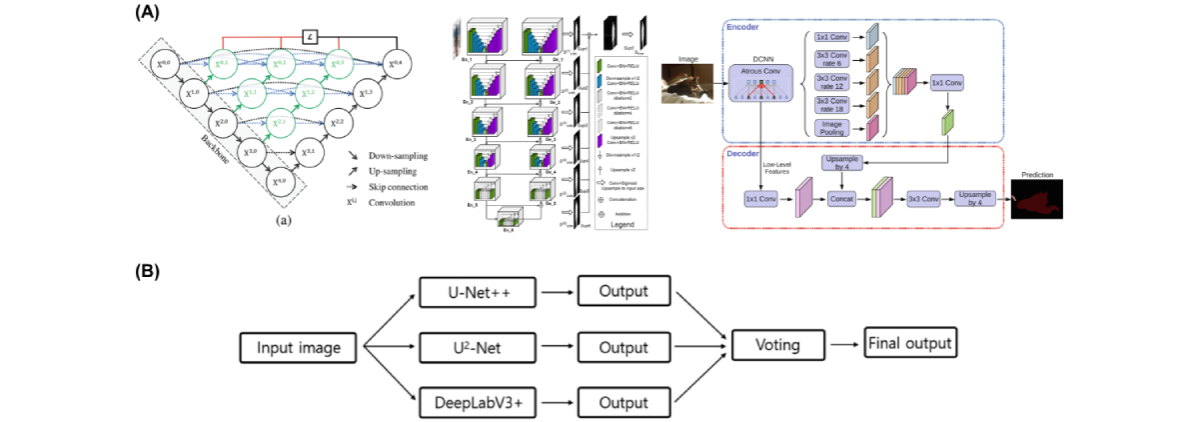
(A) Three main network architectures: U-NET++, U2-Net, and DeepLabV3+. (B) The ensemble method process. Conv: convolution; DCNN: deep convolutional neural network.

AI-QCA analysis was performed using the MPXA-2000 software. The algorithm used in MPXA-2000 was developed based on an ensemble architecture that integrated 3 neural networks for semantic segmentation (U-Net++, U2-Net, and DeepLabV3+; Figure 1) [14-16]. A hard voting classifier was also employed to improve the overall performance. A classification head for the target vessel of angiography is included at the end of each encoder in the 3 networks. In the training stage, the algorithm was trained to segment vessel mask, classify the mean vessel and side branches into 1 of the 3 types, and localize the region of interest. In an unpublished test, the average dice similarity coefficient of this segmentation algorithm was reported as 0.92, and the overall accuracy of vessel classification was 0.99. The software was authorized by the Korean Food and Drug Administration. Each image was calibrated using automatic calibration based on the isocenter calibration factor, which can be extracted from the header of the Digital Imaging and Communications in Medicine file. The best frame was automatically chosen from each video clip using densitometry. Based on 2D images, vessel segmentation, region of interest choice, vessel classification, and quantitative analysis can be performed without human intervention.

Information such as proximal and distal reference vessel diameters, minimum lumen diameter, %DS, and lesion length (LL) was provided within several seconds. Users can switch the analysis frame and modify the lesion and the segmented mask contours. Reference areas and percent area stenosis (%AS) were derived from the values estimated by AI-QCA using the following formula:



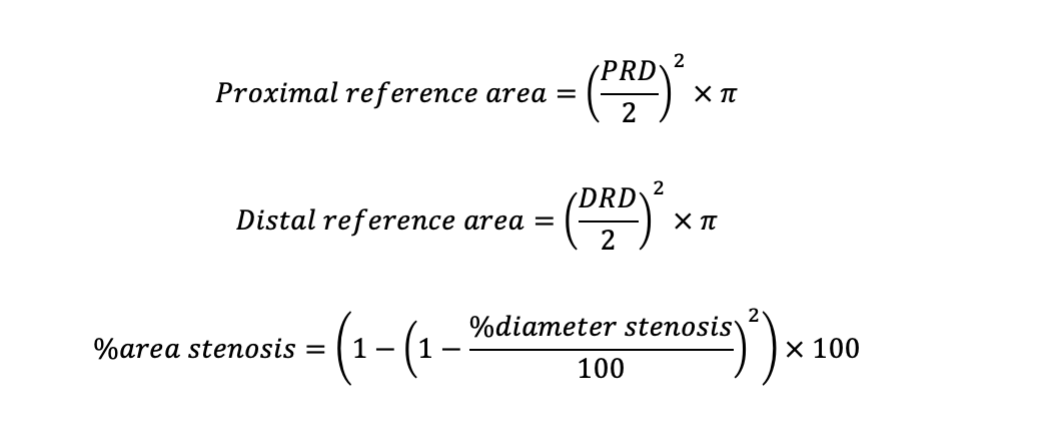



The first set of analyses was performed using the values from the fully automated AI-QCA. Data on proximal reference diameter, distal reference diameter, minimum lumen diameter, %DS, and LL were collected without any intervention from the investigators. Second, the proximal and distal borders of AI-QCA were adjusted to match those of IVUS to compare the 2 methodologies for the same coronary locations. As will be described later, geographic mismatch was the biggest cause of the discrepancy between AI-QCA and human analysis using IVUS. Third, we compared proximal-and-distal-border–adjusted AI-QCA and manual QCA.

### IVUS Analysis

IVUS was performed using 60 MHz OPTICROSS HD catheter (Boston Scientific) after intracoronary nitroglycerin administration. The proximal and distal reference areas, minimal luminal area (MLA), percent plaque burden, and LL were measured by human experts with more than 5 years of experience. IVUS analysis followed the consensus recommendations [17].

### Variables and Statistical Analysis

The study variables included proximal reference area, distal reference area, MLA, %AS, and LL. Continuous variables were expressed as mean and SD, and categorical variables as numbers and percentages. The association between the 2 methods was tested by plotting a scatter plot and measuring Pearson correlation coefficient. A correlation coefficient between 0.10 and 0.39 was considered weak, that between 0.40 and 0.69 moderate, that between 0.70 and 0.89 strong, and that between 0.90 and 1.00 very strong [18]. Bland-Altman plots were constructed to test the agreement between the 2 methods by plotting the average of the AI-QCA and IVUS measurements on the x-axis and the difference between the AI-QCA and IVUS on the y-axis. All statistical analyses were performed using R programming version 4.1.2 (The R Foundation for Statistical Computing).

## Results

Among the 50 patients initially included, AI-QCA did not work properly in 3 patients due to overlapping coronary arteries. Finally, we analyzed 54 lesions in 47 patients who underwent PCI under IVUS guidance. The baseline patient characteristics are shown in Table 1. The average age was 64.7 (SD 10.5) years. Of the 47 patients, 33 (70.2%) were male, and 24 (51%) had acute coronary syndrome. The left anterior descending, right coronary, and left circumflex arteries comprised 59.3% (n=32), 27.8% (n=15), and 13.0% (n=7) of the lesions, respectively (Table 2). Reflecting the complex study population of IVUS-guided PCI, 61.1% (n=33) of the lesions were in the bifurcation, and 35.2% (n=19) were heavily calcified lesions.

**Figure 2:**
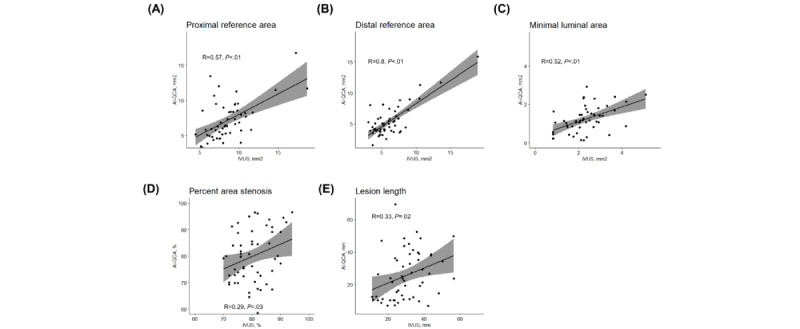
Scatter plots and Pearson correlation coefficients for (A) proximal and (B) distal reference areas; (C) minimal lumen area, (D) % area stenosis, and (E) lesion length measured by artificial intelligence–based quantitative coronary angiography (AI-QCA) and intravascular ultrasound (IVUS).

**Figure 3:**
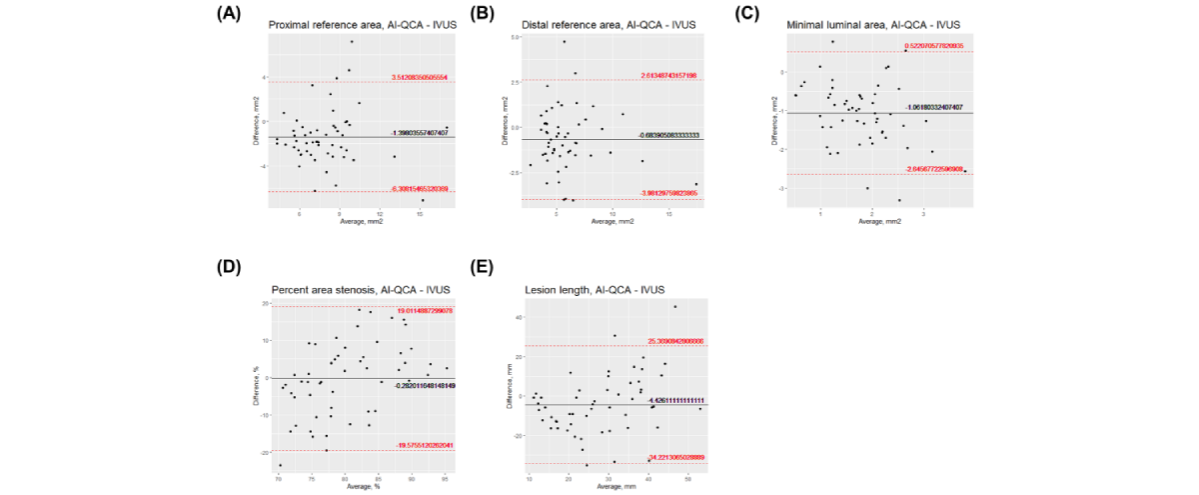
Bland-Altman plots showing the agreement between artificial intelligence–based quantitative coronary angiography (AI-QCA) and intravascular ultrasound (IVUS) for (A) proximal and (B) distal reference areas; (C) minimal lumen area, (D) % area stenosis, and (E) lesion length. The x-axis is the average of variables measured by AI-QCA and IVUS, and the y-axis is the difference of AI-QCA minus IVUS.

First, we compared the values from the fully automated AI-QCA with IVUS. Figure 2 shows the scatter plots of the study variables. Measurements for the reference and lesion areas showed moderate to strong correlations between the 2 modalities (correlation coefficients of 0.57 for proximal reference, 0.80 for distal reference, and 0.52 for MLA; *P*<.001). Meanwhile, %AS and LL showed a weaker correlation (correlation coefficient of 0.29 and *P*=.03 for %AS and correlation coefficient of 0.33 and *P*=.02 for LL). The Bland-Altman plots for agreement between the AI-QCA and IVUS measurements are shown in Figure 3. The AI-QCA measured reference areas smaller than human observers using IVUS with no systematic proportional bias. Most observations were within an error margin of 4 mm^2^. The AI-QCA tended to measure LL shorter than human observers with IVUS.

%AS showed the weakest correlation among the variables. We divided the patients into 2 groups, with high and low agreement in %AS. The low agreement group, which is a group with a difference of more than 10% of %AS measured by AI-QCA and IVUS, had numerically lower heavy calcified lesions (Table S1 in Multimedia Appendix 1). The difference of less than 10% of %AS does not affect the decision to perform PCI.

**Figure 4:**
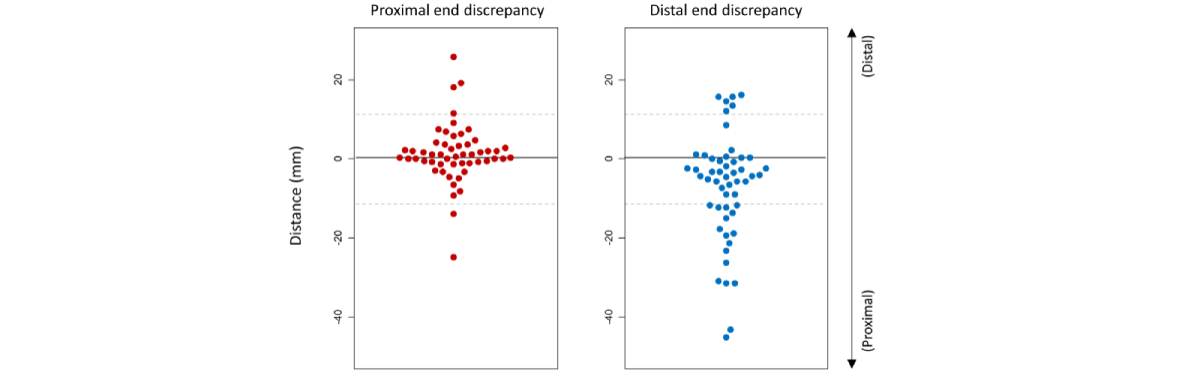
Geographic mismatch in lesion identification between artificial intelligence–based quantitative coronary angiography (AI-QCA) and human observers under intravascular ultrasound (IVUS) guidance. The reference point of y-axis is the proximal and distal margin determined by IVUS. A positive value means the margin determined by AI-QCA is more distal than that by IVUS.

The weak correlation for LL was driven by the geometric mismatch of lesion identification between the human observers and the AI-QCA (Figure 4). The proximal border identified by AI-QCA was mostly within 10 mm of that identified by human observers in 48 (88.7%) lesions. However, the distal border showed a greater discrepancy—AI-QCA identified the distal border more proximally than human observers guided by IVUS. As a result, AI-QCA generally estimated a shorter LL compared with IVUS.

**Figure 5:**
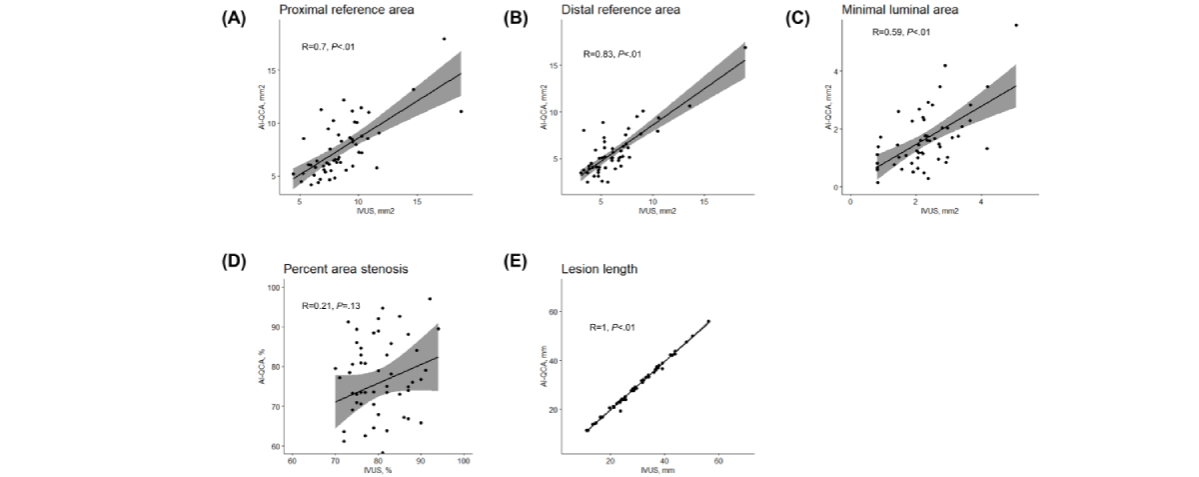
Scatter plots and Pearson correlation coefficients for (A) proximal and (B) distal reference areas; (C) minimal lumen area, (D) % area stenosis, and (E) lesion length measured by artificial intelligence–based quantitative coronary angiography (AI-QCA) and intravascular ultrasound (IVUS) after adjusting proximal and distal margins.

Next, we adjusted the proximal and distal margins detected by AI-QCA to align with those determined by human observers under IVUS guidance. The proximal and distal reference areas and MLA showed numerically greater correlation coefficients than the initial analysis (0.70 for proximal reference area, 0.83 for distal reference area, and 0.59 for MLA; *P*<.001), while %AS still showed weak correlation (0.21, *P*=.13; Figure 5). Bland-Altman plots (Figure S1 in Multimedia Appendix 1) show that the mean differences in reference areas and MLA between AI-QCA and IVUS were smaller than those between fully automated AI-QCA and IVUS.

AI-QCA showed strong correlation with manual QCA except proximal reference diameter (Figure S2 in Multimedia Appendix 1). Figure S3 in Multimedia Appendix 1 shows the correlation coefficients measured by IVUS and manual QCA. Correlation coefficients between AI-QCA and IVUS were similar to those between manual QCA and IVUS (0.70 vs 0.76 for proximal reference area, 0.83 vs 0.82 for distal reference area, 0.59 vs 0.59 for MLA, 0.21 vs 0.22 for %AS, and 1.00 vs 0.98 for LL).

**Figure 6:**
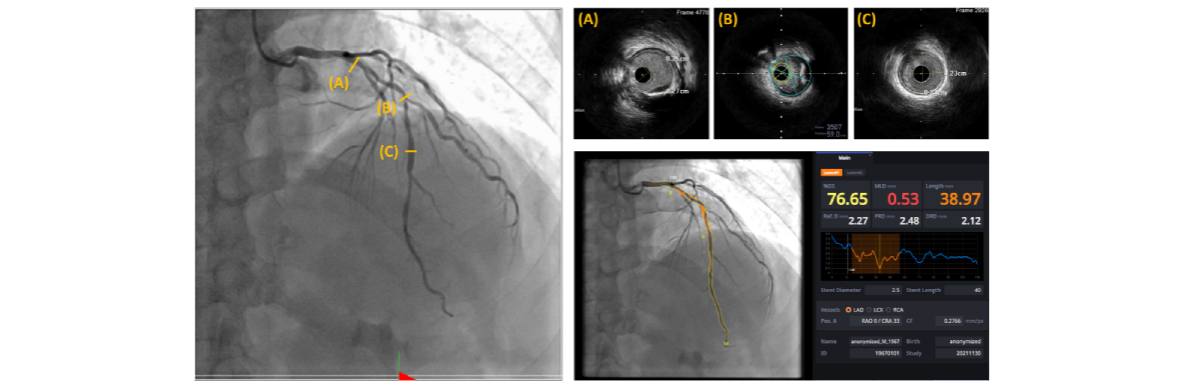
A representative case in which artificial intelligence–based quantitative coronary angiography (AI-QCA) showed a good correlation with intravascular ultrasound (IVUS) observation. %DS: percent diameter stenosis; DRD: distal reference diameter; LAD: left anterior descending artery; LCX: left circumflex artery; MLD: minimal luminal diameter; PRD: proximal reference diameter; RCA: right coronary artery; Ref.D: reference diameter.

**Figure 7:**
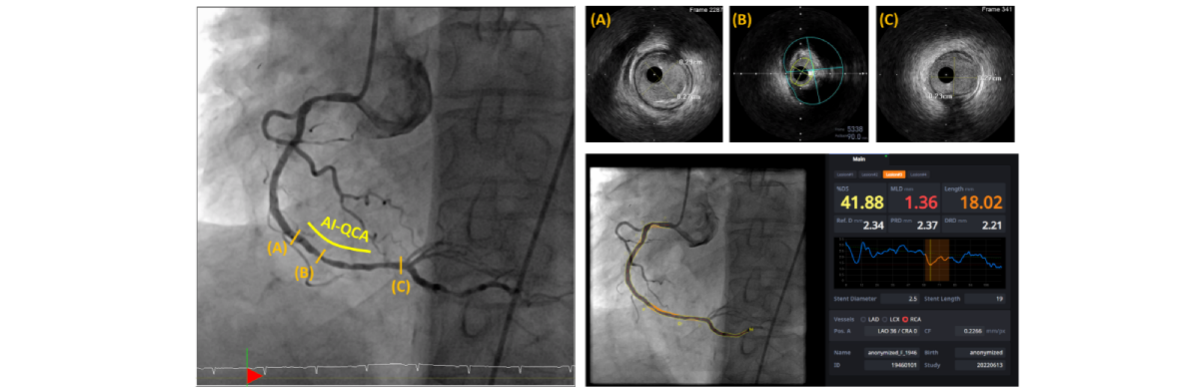
A representative case in which artificial intelligence–based quantitative coronary angiography (AI-QCA) measured lesion length shorter than intravascular ultrasound (IVUS). AI-QCA identified mild atherosclerotic lesion at distal as normal. %DS: percent diameter stenosis; AI-QCA: artificial intelligence–based quantitative coronary angiography; DRD: distal reference diameter; LAD: left anterior descending artery; LCX: left circumflex artery; MLD: minimal luminal diameter; PRD: proximal reference diameter; RCA: right coronary artery; Ref.D: reference diameter.

Figure 6 shows a representative case in which AI-QCA showed a good correlation with IVUS. LL was estimated to be 39.0 mm with AI-QCA and 37.1 mm with IVUS. %DS by AI-QCA was 76.7%, and plaque burden on IVUS was 78%. Figure 7 shows another representative case in which AI-QCA identified the distal border more proximally than IVUS. AI-QCA separated the distal right coronary artery lesion into 2 segments, which was considered a single continuous lesion under IVUS guidance.

**Table 1:** Patient characteristics.

Variables			Values
Age (years), mean (SD)			64.7 (10.5)
**Sex, n (%)**			
	Male	33 (70.2)	
	Female	14 (29.8)	
**Smoking history, n (%)**			
	Nonsmoker	24 (51.1)	
	Previous smoker	10 (21.3)	
	current smoker	13 (27.7)	
**Clinical diagnosis, n (%)**			
	Myocardial infarction	12 (25.5)	
	Unstable angina	12 (25.5)	
	Stable angina	16 (34.0)	
	Heart failure, others	7 (14.9)	
**Underlying disease, n (%)**			
	Hypertension	29 (61.7)	
	Diabetes mellitus	27 (57.4)	
	Dyslipidemia	15 (31.9)	
	Chronic kidney disease	6 (12.8)	
	Stroke (ischemic and hemorrhagic)	6 (12.8)	
	Previous coronary artery disease	6 (12.8)	
**Laboratory findings, mean (SD)**			
	Hemoglobin (g/dL)	13.5 (2.0)	
	Fasting glucose (mg/dL)	157.0 (65.4)	
	Creatinine (mg/dL)	1.7 (2.5)	
	Total cholesterol (mg/dL)	159.2 (50.2)	
	Triglyceride (mg/dL)	150.7 (83.7)	
	HDL^a^-cholesterol (mg/dL)	37.9 (9.2)	
	LDL^b^-cholesterol, mg/dL	103.8 (53.5)	
	Hemoglobin A_1c_ (%)	6.8 (1.9)	

^a^HDL: high-density lipoprotein.

^b^LDL: low-density lipoprotein.

**Table 2:** Lesion characteristics.

Characteristics		Values, n (%)
**Location**		
	Left anterior descending artery	32 (59.3)
	Right coronary artery	15 (27.8)
	Left circumflex artery	7 (13.0)
	Bifurcation	33 (61.1)
	Heavy calcified lesion	19 (35.2)
	Ostial disease	8 (14.8)
	Long lesion	9 (16.7)
**Disease extent**		
	One-vessel disease	16 (29.6)
	Two-vessel disease	20 (37.0)
	Three-vessel disease	18 (33.3)

## Discussion

### Principal Findings

In this study, we found that the AI-QCA showed a moderate to strong correlation with human assessment guided by IVUS. The reference vessel size and stenosis severity were moderately correlated. Geographic mismatch was present in certain cases, indicating that there was discrepancy in the proximal or distal lesion margins between AI-QCA and IVUS. Discrepancies were frequently observed at distal margins.

### Strengths of This Study

The application of AI is expanding in various fields of medicine. Machine learning and computer vision were introduced first and have proven their roles in radiology and pathology [19,20]. The adoption of medical AI has been slow in cardiology, partly due to the 3D nature and video format of cardiology images. Several recent studies have tested the application of AI in echocardiography, owing to recent advances in computing power and machine learning algorithms [21,22]; however, research has been scarce in the field of coronary angiography [23,24].

### Interpretation of Results

The observation of this study, that AI-QCA underestimates vessel size compared to IVUS, is in line with the findings from previous studies. Studies have shown that QCA measurements are usually smaller than intracoronary imaging including optical coherence tomography and IVUS [25]. A postmortem study also found that intracoronary imaging overestimates the lumen area compared with the histomorphometry [26]. IVUS measurements are generally larger than those obtained using optical coherence tomography.

Since there are scarce data on AI-QCA, the relationship between coronary artery calcification and AI-QCA measurements is not well known. In this study, heavy calcification may have affected the AI-QCA measuring %AS, although not statistically significant. The finding that AI-QCA estimates an LL shorter than IVUS does can be partly explained by the tomographic images provided by IVUS. Observers can identify mild atherosclerotic changes with IVUS that appear normal on the angiography [27]. It is well known that physicians tend to use longer and larger stents during IVUS-guided PCI [28].

This study showed a relatively good correlation with MLA, but a weaker correlation with %AS. One possible reason for this is that positive remodeling is reflected in IVUS. Positive remodeling and vessel wall expansion occur during the early phase of atherosclerosis to maintain lumen size despite plaque accumulation. %DS is calculated only based on the reference diameter assumed by the interpolation of proximal and distal normal-looking segment diameters. In addition, reference diameters can be underestimated because proximal and distal reference segments may not be free of atherosclerosis, as discussed above. The plaque burden assessed by IVUS is greater than the %AS by QCA [29]. This study population represented complex coronary diseases—61% with bifurcation and 35% with heavily calcified lesions. A previous study also found intercore lab variability in the analysis of %DS for bifurcation lesions [30]. In this study, we calculated %AS from %DS from 2D images using the previously mentioned equation (Methods section). It is anticipated that 3D QCA may improve the accuracy of lesion severity.

### Clinical Implication

Physicians performing PCI require considerable experience to accurately assess the characteristics of coronary arteries and the burden of atherosclerotic plaques. IVUS is the most commonly used intravascular imaging tool for optimizing coronary stenting [31,32]. This study showed a moderate to strong correlation between an AI-QCA that automatically analyzed 2D angiography images and IVUS analysis. Physicians could consult AI-QCA during PCI and consider a one-step larger diameter stent, as this study suggested AI-QCA tended to underestimate reference vessel area. In addition, physicians should be aware that AI-QCA may underestimate mildly atherosclerotic lesion as normal.

The AI-QCA tested in this study was based on deep learning algorithms intended to mimic the QCA process by human experts. This tool may be helpful for interventional cardiologists who feel less confident in determining stent size based on angiography alone when intravascular imaging is not available.

### Limitations

This study is not free from limitations. First, this was a single-center study with a small sample size; therefore, caution should be exercised when extrapolating the findings to other studies. Since this study population represents a significant coronary disease that requires complex coronary intervention, the findings cannot be extrapolated to mild to intermediate coronary lesions. While the software was developed as a real-time coronary intervention assistance tool, the AI-QCA was performed separately because of the retrospective nature of this study. Second, IVUS was performed after predilatation in some cases because of the delivery failure of IVUS catheter, which may lead to larger MLA than the initial angiography. Third, even though the qualitative component of coronary artery, such as calcification or tortuosity, is the important value for clinicians to make the right decision, AI-QCA cannot assess the characteristics of coronary arteries. Correlation with IVUS measurements may not be a gold standard indicator for evaluating the accuracy of AI-QCA.

Future studies are required to address the utility of the software in real world clinical practice.

### Conclusion

In this study, AI-QCA showed moderate to strong correlation accuracy compared with IVUS measurements in patients with coronary artery disease who underwent coronary intervention. This study provides supporting evidence that the AI-QCA can be safely used in clinical practice. Automated real-time analysis of coronary angiography may help practitioners make clinical decisions with greater confidence. Further prospective studies are needed to confirm AI-QCA’s clinical utility and safety.

## Appendix

Multimedia Appendix 1 Table S1. Lesion characteristics of high and low agreement groups in percent area stenosis. Figure S1. Bland-Altman plots of proximal and distal reference areas, minimal luminal area (MLA), percent area stenosis (%AS), and lesion length (LL). The x-axis is the average of variables measured by artificial intelligence–based quantitative coronary angiography (AI-QCA) and intravascular ultrasound (IVUS); the y-axis is the difference value of AI-QCA minus IVUS. Figure S2. Scatter plots and Pearson correlation coefficients for (A) proximal and (B) distal reference diameters; (C) minimum lumen diameter, (D) % diameter stenosis, and (E) lesion length measured by AI-QCA and manual quantitative coronary angiography (QCA). Figure S3. Scatter plots and Pearson correlation coefficients for (A) proximal and (B) distal reference areas; (C) minimal lumen area, (D) % area stenosis, and (E) lesion length measured by IVUS and manual QCA.

## Abbreviations

%AS: percent area stenosis
%DS: percent diameter stenosis
AI: artificial intelligence
AI-QCA: artificial intelligence–based quantitative coronary angiography
IVUS: intravascular ultrasound
LL: lesion length
MLA: minimal luminal area
PCI: percutaneous coronary intervention
QCA: quantitative coronary angiography

## Data Availability

The data sets generated and analyzed during the current study are available from the corresponding author on reasonable request.
