# Supplementary material for: Accuracy of Artificial Intelligence–Based Automated Quantitative Coronary Angiography Compared to Intravascular Ultrasound: Retrospective Cohort Study
Source: JMIR Cardio. 2023 Apr 26;7:e45299. doi: 10.2196/45299 (PMC10173041; doi:10.2196/45299)
Supplement: Multimedia Appendix 1 [file cardio_v7i1e45299_app1.docx]

| **Table S1. Lesion characteristics of high and low agreement groups in percent area stenosis** | | | |
| --- | --- | --- | --- |
|  | High agreement group  (%AS difference<10%)  n=36 | Low agreement group  (%AS difference>10%)  n=18 | p-value |
| Location  Left anterior descending artery  Right coronary artery  Left circumflex artery | 21 (58.3%)  10 (27.8%)  5 (13.9%) | 11 (61.1%)  5 (27.8%)  2 (11.1%) | 0.957 |
| Bifurcation | 22 (61.1%) | 11 (61.1%) | 1.000 |
| Heavy calcified lesion | 15 (41.7%) | 4 (22.2%) | 0.268 |
| Ostial disease | 5 (13.9%) | 3 (16.7%) | 1.000 |
| Long lesion | 6 (16.7%) | 3 (16.7%) | 0.495 |
| Disease extent  1 vessel disease  2 vessel disease  3 vessel disease | 9 (25.0%)  15 (41.7%)  12 (33.3%) | 7 (38.9%)  5 (27.8%)  6 (33.3%) | 0.495 |
| More than 1 stent used | 5 (13.9%) | 3 (16.7%) | 1.000 |


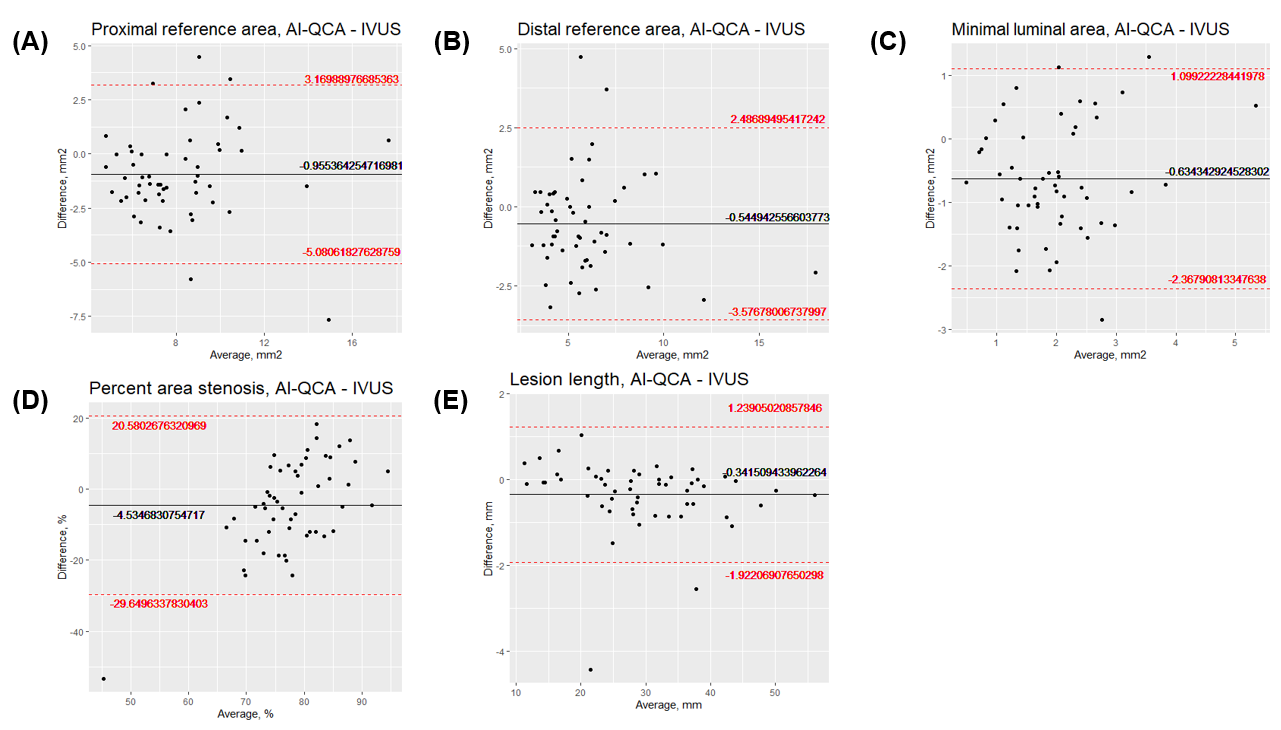
 **Figure S1.** Bland-Altman plots of proximal and distal reference areas, MLA, %AS, and LL. X axis is the average of variables measured by AI-QCA and IVUS, y axis is the difference value of AI-QCA minus IVUS.


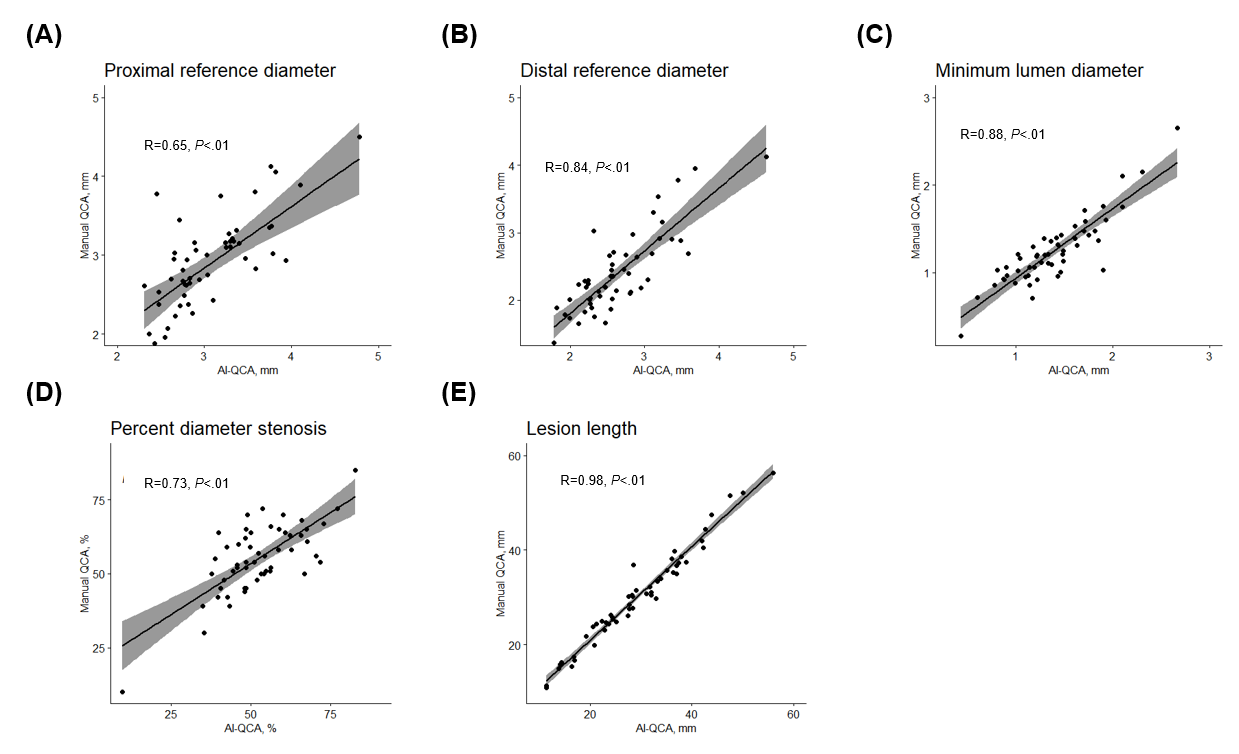


Figure S2. Scatter plots and Pearson’s correlation coefficients for (A) proximal and (B) distal reference diameters, (C) minimum lumen diameter, (D) % diameter stenosis, and (E) lesion length measured by artificial intelligence-based quantitative coronary angiography (AI-QCA) and manual quantitative coronary angiography (QCA).


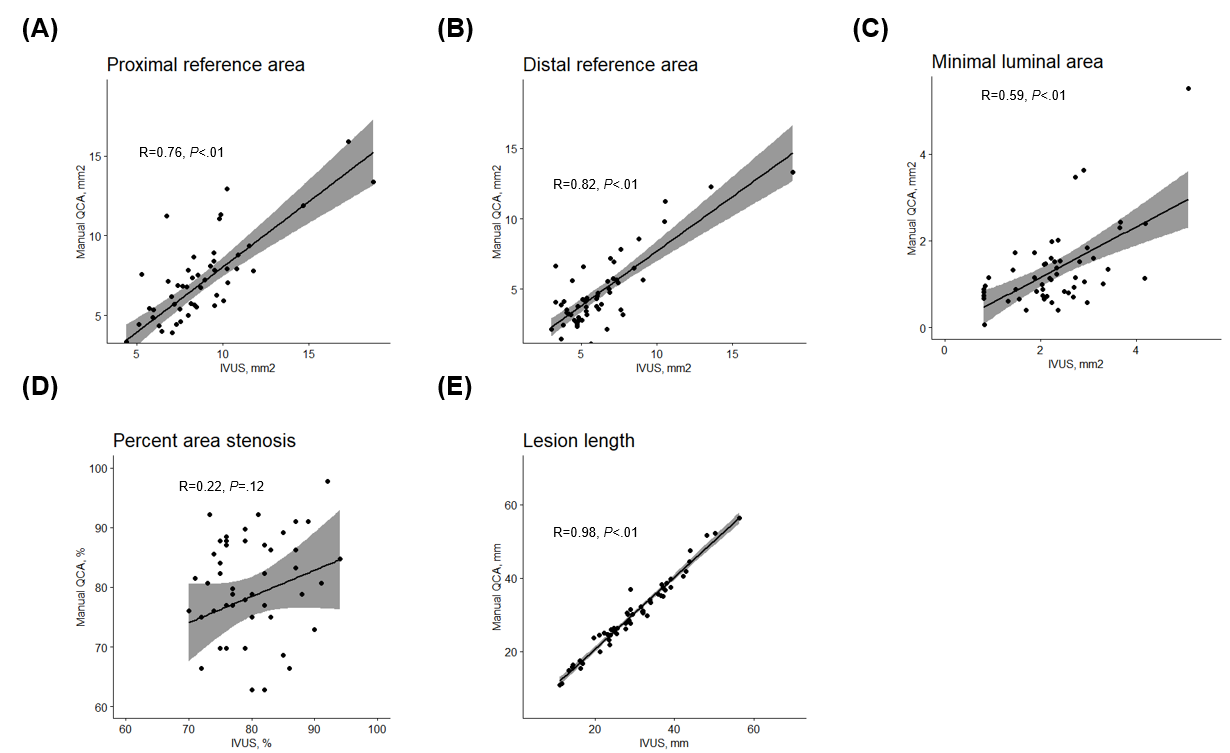


Figure S3. Scatter plots and Pearson’s correlation coefficients for (A) proximal and (B) distal reference areas, (C) minimal lumen area, (D) % area stenosis, and (E) lesion length measured by intravascular ultrasound (IVUS) and manual quantitative coronary angiography (QCA).
